# The Public Relations of Post-Mortem Examinations

**Published:** 1915-03-06

**Authors:** 


### The Hospital

The Workers' Newspaper of Administrative Medicine and Institutional Life, Administration, National Insurance and Health.

No. 1498, Vol. LVII. SATURDAY, MARCH 6, 1915. PRICE ONE PENNY.

### THE PUBLIC RELATIONS OF POST-MORTEM EXAMINATIONS.

Except in professional circles, the practice of *post-mortem* examination repels rather than invites attention, and the general and conventional attitude is to ignore it. Yet in certain of its relations it is closely concerned with urgent public interests. That there are certain circumstances in which it is necessary no one denies. But, while this is admitted, the admission is made with some measure of regret, and the proceeding is viewed as unhappily inevitable in order to settle, perhaps for legal purposes, some particular and individual issue. A proposal to multiply such examinations runs the risk of public reprobation. In so far as this attitude of mind is an expression of a desire that the dead body should be treated with respect, it is worthy of reverence; and to describe it as mere sentiment is not to empty it of value. At the same time, it is well to recall certain practical necessities. Further, there exists the possibility of an education which may teach both that the satisfaction of these necessities is essential to human welfare, and that *post-mortem* examination and respect for the dead are far from being mutually inconsistent. On this latter issue it is, perhaps, sufficient to recall the custom followed in Royal families, which, though adopted mainly for dynastic and national motives, disposes once for all of the suspicion of any failure of reverence. To attempt to push sentiment out of life would be a task both vain and foolish. But it is possible to elevate and to refine sentiment, and to confer on it the wider vision which sees that individual dignity is heightened, and not lowered, when contributing to the general interest. An attempt to lead public opinion to this level is readily capable of justification.

The immediate purpose of a *post-mortem* examination is, of course, to make certain the cause of the death of an individual. As in the majority of cases this cause is known from the symptoms which distinguished the last and fatal illness, the conclusion is readily accepted that such examination is rarely necessary. In this conclusion there is some measure of truth, though it must be remembered that amidst the complexities of a serious illness mistaken diagnosis is a possibility, and that detection of the error may never be secured unless a *post-mortem* examination is permitted. To detect error in these circumstances may bring little comfort to those immediately concerned, but to the practitioner the experience is an impressive one. Valuable in itself, it has this larger worth, that it is likely to protect him from the same and kindred errors in future, and here the *post-mortem* examination touches general interests. The alternative implies at least the chance of repeated mistakes, and it is hardly too much to say that a true understanding of the position, so far from objecting to an examination, would insist upon it, at least in any case attended by any appreciable degree of obscurity or doubt. To a considerable extent the education of the public in this direction depends on the tact and discretion of individual medical practitioners, who, realising the possible educational gain, are able to carry the same conviction to other minds, and to show that what is proposed involves neither lack of sympathy nor mere purposeless curiosity.

To pass now beyond individual interests, the practice of *post-mortem* examination has to be recognised as contributing an element which is essential both to efficient medical education and to the advance of medical knowledge. The facts of pathological change must be made real to the student, and must be correlated in his mind with the symptoms of disease which he has observed in the living patient. Here, indeed, is the very basis of confident knowledge in relation alike to diagnosis and to treatment. Though pathological anatomy may be viewed as a record of wreck and disaster, it is a lesson which must be learned before the possibility of appreciating the processes responsible for the catastrophe can even be divined. And to appreciate these processes is the first step towards the capacity to prevent or arrest them in future instances. Moreover, the message of the dead body is not limited to an announcement of the changes responsible for the fatal issue. On the contrary, much has been learned both of the functions of organs and of their ability to resist the processes of disease from examinations which were conducted primarily for other ends. In this latter sense the teaching derived from *post-mortem* anatomy has been by no means wholly of a gloomy character. To take but a single instance, the relatively cheerful outlook now provided for tuberculosis owes its existence very largely to the evidence of frequent healed lesions provided by *post-mortem* examination. Thus it is from *post-mortem* examinations properly conducted that there arises a confident acquaintance with the reactions of the tissues under abnormal circumstances. This is the foundation upon which medical education, if it is to be fruitful, must rest, and as efficient medical education concerns in a high degree the welfare of the community, there are public interests which demand that opportunities for the practice of *post-mortem* examination ought to be encouraged.

